# Pooled cohort equations heart failure risk score predicts cardiovascular disease and all-cause mortality in a nationally representative sample of US adults

**DOI:** 10.1186/s12872-020-01485-2

**Published:** 2020-04-25

**Authors:** Alexander C. Razavi, Kaitlin S. Potts, Tanika N. Kelly, Jiang He, Camilo Fernandez, Marie Krousel-Wood, Amanda H. Anderson, Joshua Bundy, Seamus P. Whelton, Roger S. Blumenthal, Donald Lloyd-Jones, Lydia A. Bazzano

**Affiliations:** 1 Department of Epidemiology, Tulane University School of Public Health and Tropical Medicine, 1440 Canal Street, Suite 2000, New Orleans, Louisiana, 70112 USA; 2 Department of Medicine, Tulane University School of Medicine, New Orleans, Louisiana, USA; 3 The Ciccarone Center for the Prevention of Cardiovascular Disease, Johns Hopkins University School of Medicine, Baltimore, MD USA; 4 Department of Preventive Medicine, Northwestern University Feinberg School of Medicine, Chicago, IL USA

**Keywords:** Heart failure, Risk, Cardiovascular diseases, Mortality, Primary prevention, NHANES, Social determinants of health, Epidemiology, Blood pressure, Electrocardiography

## Abstract

**Background:**

Heart failure (HF) represents an accumulated burden of systemic vascular damage and is the fastest growing form of cardiovascular disease (CVD). Due to increasing HF-attributable mortality rates, we sought to assess the association of the new 2019 Pooled Cohort equations to Prevent Heart Failure (PCP-HF) risk score with CVD and all-cause mortality.

**Methods:**

We linked data for 6333 black and white men and women aged 40–79 years, whom underwent electrocardiographic examination from the Third National Health and Nutrition Exam Survey, to National Death Index record matches. Sex- and race-specific PCP-HF risk scores were calculated using data on age, smoking, body mass index, systolic blood pressure, total cholesterol, HDL-cholesterol, fasting blood glucose, QRS complex duration, and antihypertensive and/or glucose-lowering medications. Cox regression estimated hazard ratios for the association of the PCP-HF risk score with CVD and all-cause mortality.

**Results:**

Individuals were on average 54.9 years old (51.7% women, 25.4% black) and the median 10-year HF risk was 1.6% (Q1 = 0.5, Q3 = 4.8). There were 3178 deaths, 1116 from CVD, over a median follow-up time of 22.3 years. Black women had a higher 10-year HF risk compared to white women (2.1% vs. 1.1%; *p* < 0.01), while no significant difference was observed in predicted HF risk between black men and white men (2.3% vs. 2.1%, *p* = 0.16). A two-fold higher PCP-HF risk score was associated with a significant 58% (HR = 1.58; 95% CI, 1.48–1.70; *p* < 0.0001) and 38% (HR = 1.38; 95% CI, 1.32–1.46; p < 0.0001) greater risk of CVD and all-cause mortality, respectively.

**Conclusion:**

The PCP-HF risk score predicts CVD and all-cause mortality, in addition to the 10-year risk of incident HF among white and black men and women. These results underline the expanded utility of the PCP-HF risk score and suggest that its implementation in the clinical and population health settings may improve primary CVD prevention in the United States.

## Background

Heart failure (HF) is the fastest growing form of cardiovascular disease (CVD) nationally and globally, as its prevalence is projected to increase by 46% over the next decade in the United States [1–3]. The HF-attributable mortality rate has increased by over 20% since 2011 [4] and the median survival time after an initial HF diagnosis is as low as 2.1 years [5], differing by race, ethnicity, and socioeconomic status [6]. Because HF is primarily a non-reversible disease, primary prevention is a key strategy to reducing population morbidity and mortality. Similar to other forms of CVD, HF may be largely preventable through lifestyle approaches and modification of common risk factors, including hypertension, dyslipidemia, obesity, and type II diabetes [7].

In an effort to improve HF risk stratification and prevention, the Pooled Cohort equations to Prevent HF (PCP-HF) risk score was developed and validated in 2019 using combined individual-level data from five diverse population-based cohorts to estimate the 10-year risk for incident HF [8]. Though a variety of HF risk scores exist [9–12], the PCP-HF contains parameters that are easily obtained in a primary care setting, and was developed with information from younger adults as well black men and women, in contrast to current risk prediction tools.

Data from both observational studies [13] and randomized controlled trials [14] indicate that HF is a heterogeneous disease, and suggests that further effort must be directed more upstream on the causal disease pathway, prior to clinical HF manifestation. While the PCP-HF risk score has successfully been validated for HF risk prediction in black and white adults across cohort studies [8], it is not known whether the PCP-HF risk score predicts future CVD- and/or all-cause mortality. Likewise, differences in the estimated 10-year risk for incident HF according to level of income, education, marital status, and living environment remain ill-defined. Information on the social determinants that may help characterize different patterns of HF in vulnerable populations may inform early preventive action against HF onset, and identify epidemiologic and clinically pertinent differences in predicted HF risk among key sociodemographic populations. To our knowledge, this is the first investigation of the predictive and discriminative ability of the PCP-HF risk score in the general United States population using the National Health and Nutrition Examination Survey (NHANES). We estimated the 10-year risk for incident HF and assessed the association of the PCP-HF risk score with CVD- and all-cause mortality in black and white women and men living in the United States.

## Methods

The NHANES uses a complex, stratified, multistage probability cluster sampling design to select a representative sample of noninstitutionalized citizens living in the United States. The purpose of these surveys is to describe the epidemiology of multiple conditions and health status of Americans. The baseline examinations for NHANES III occurred from 1988 to 1994 and detailed methods regarding data collection have been previously described [15]. National Death Index searches, made available through public datasets at the National Center for Health Statistics, were linked to NHANES III participants and provided mortality follow-up data [16]. A total of 8561 men and women aged 40–79 years had available electrocardiogram data with measures of QRS complex duration. After excluding individuals missing data necessary to derive PCP-HF risk scores (*n* = 1775), and individuals with self-reported HF (*n* = 453), 6333 persons remained in the analysis. Among these 6333 persons were 2302 white men, 2421 white women, 754 black men, and 856 black women.

Standardized household interviews were used to collect information on sociodemographic variables, including sex, race, medical/medication history, income and education level, marital status, and area of residence. Physical examinations were performed at a mobile examination center. Blood pressure was measured in triplicate using a mercury sphygmomanometer by trained physicians after study participants had undergone 5 min of rest [17]. A twelve-lead electrocardiogram was performed with study participants still, supine and breathing normally. Electrocardiogram data and tracings were uploaded to and stored on a Marquette MAC 12 unit for accurate analysis [18]. An electronic digital scale and steel tape measure were used to measure weight and height respectively [19]. Total serum cholesterol was measured enzymatically, while HDL-cholesterol was measured after precipitation of other lipoproteins with a polyanion/divalent cation mixture. Glucose was measured enzymatically in plasma in the fasted state via the hexokinase method [20].

Sex- and race-specific 10-year PCP-HF risk scores were calculated via equations derived by Khan et al. [8]. Individual coefficient values were calculated for each sex and race group using data on age, treated or untreated systolic blood pressure, smoking status, treated or untreated fasting glucose, total cholesterol, HDL-cholesterol, body mass index, and QRS complex duration. Coefficient values were summed to generate an individual coefficient value for each study participant. Both the individual coefficient values and mean coefficient values were then used to estimate the 10-year risk of a HF event through the following formal equation as previously described [8]: 1-S_o_^e(individual coefficient value – mean coefficient value)^. Further explanation of risk score components and calculations are provided in Supplemental Table 1.

CVD mortality and all-cause mortality were defined as the primary and secondary outcome, respectively, for this observational study. CVD mortality was defined using the *International Classification of Diseases*-10th Revision (ICD-10) codes, I00-I09, I11, I13, I20-I51, I60-I69 [21].

We described the sample according to United States population weights, using means for normally distributed continuous variables, medians for non-normally distributed continuous variables, and percentages for categorical variables in the total sample. Weighted standard errors, interquartile ranges, and ranges were presented as measures of dispersion when appropriate. Normality of continuous variables was assessed via the Kolmogorov-Smirnov test. The PCP-HF risk score was transformed to the log_2_ scale to normalize its distribution prior to multivariable analysis. This method of transformation was chosen for interpretation purposes, as a one unit increase on the log_2_ scale corresponds to a doubling, or two-fold increase in the transformed variable. The Student’s t-test and Wilcoxon signed-rank test were used to assess differences in normally and non-normally distributed continuous variables, respectively. Differences between categorical variables were evaluated using Pearson’s chi-square test. Survival curves by quartile of PCP-HF risk score were plotted and compared using the log-rank test to assess differences in CVD and all-cause mortality among risk quartiles. We conducted race- and sex-stratified analyses when appropriate.

Crude CVD-attributed death rates were calculated per PCP-HF risk score quartile as the number of CVD deaths divided by the sum of follow-up years per 1000 person-years for each five-year age strata. Age-adjusted CVD mortality rates per quartile of PCP-HF risk score were calculated by standardizing to the age-distribution of the total sample. Standard errors of the age-adjusted rates were estimated by dividing the age-adjusted rate by the square-root of the number of events [22]. These were used to calculate 95% confidence intervals (CIs) and compare age-adjusted CVD mortality rates across PCP-HF risk score quartile. Trend in age-adjusted mortality across PCP-HF risk score quartiles was tested using the median quartile value for each individual observation.

The association of the PCP-HF risk score with CVD and all-cause mortality was assessed using Cox proportional hazards regression models. The proportional hazards assumptions were found to be appropriate. Potential confounding variables were chosen a priori based on established associations with HF and CVD mortality [23]. We estimated the association between the PCP-HF and death due to CVD and all-causes using three models: 1) unadjusted; 2) adjusted for age, sex, and race; 3) adjusted for variables in model 2 plus previous history of myocardial infarction, annual income level (below $25,000 versus $25,000–$50,000 versus more than $50,000), education status (high school education or lower versus post high school education), living environment (urban versus non-urban), and living status (living alone versus living with a partner). Discrimination ability of the models to differentiate between events and non-events was evaluated using C-statistics. C-statistics were computed and presented as a measure of discrimination in the context of prediction accuracy of the model, as previously described [24, 25]. Statistical analyses were performed using R and SAS 9.3 (SAS Institute, Cary, NC). Sample weights and complex survey design effect were accounted for in all analyses (R package “survey”; SAS procedures surveymeans, surveyfreq, surveyphreg, surveyreg, and surveylogistic). All hypothesis tests were 2-sided. We used an alpha threshold of 0.05 for detecting differences in descriptive statistics and a Bonferroni-corrected alpha threshold of 0.025 (0.05/2) for proportional hazard regression models (primary outcome = CVD mortality; secondary outcome = all-cause mortality).

## Results

Table 1 presents baseline characteristics and mortality data according to United States population sampling weights for the 6333 NHANES participants (mean age: 54.9 years; 3277 women; 1610 black) included in the analyses. The study sample, on average, was overweight (BMI = 27.4 + 0.1 kg/m^2^) and had elevated systolic blood pressure (129.7 + 0.4 mmHg) and cholesterol (total cholesterol = 216.7 + 0.9 mg/dL). A total of 3178 individuals died during a median follow-up time of 22.3 years, and 1116 deaths were attributed to CVD (Supplemental Table 2). There were significantly more men, individuals living alone, and individuals with low educational attainment per increasing PCP-HF risk score quartile. Additionally, we observed a higher proportion of CVD-attributable and total deaths when comparing trends from the lowest to highest PCP-HF risk score quartiles.

**Table 1:** Characteristics of 6333 Black and White NHANES III Participants Without Heart Failure

Variable*†	All (***n*** = 6333)	PCP-HF Q1 (***n*** = 1583)	PCP-HF Q2 (n = 1583)	PCP-HF Q3 (***n*** = 1584)	PCP-HF Q4 (n = 1583)	***P***-value for Trend
***Sociodemographic & Lifestyle***						
Age, years, mean	54.9 (0.3)	44.9 (0.2)	53.1 (0.3)	62.4 (0.4)	68.4 (0.4)	< 0.0001
Female, %	52.3 (0.7)	62.6 (1.2)	49.9 (1.6)	48.8 (1.8)	39.6 (1.8)	< 0.0001
Black, %	9.2 (0.6)	5.8 (0.5)	11.6 (0.8)	11.9 (0.9)	9.1 (1.0)	0.08
Current Smoking, %	24.3 (1.0)	18.0 (1.6)	32.1 (2.0)	23.9 (1.5)	25.3 (2.0)	0.19
Living Environment, %						0.15
Urban	46.1 (4.6)	47.4 (5.1)	47.2 (4.9)	45.3 (4.4)	42.9 (4.8)	
Non-Urban	53.9 (4.6)	52.6 (5.1)	52.8 (4.9)	54.7 (4.4)	57.1 (4.8)	
Living Status, %						< 0.001
Living Alone	26.7 (0.9)	23.3 (1.7)	25.0 (1.9)	29.7 (1.7)	32.6 (1.7)	
Living with Partner	73.3 (0.9)	76.7 (1.7)	75.0 (1.9)	70.3 (1.7)	67.4 (1.7)	
Educational Attainment, %						< 0.0001
High-School Education or Below	60.7 (1.6)	47.1 (2.3)	61.9 (2.2)	71.1 (1.9)	73.6 (2.0)	
Post High-School Education	39.3 (1.6)	52.9 (2.3)	38.1 (2.2)	28.9 (1.9)	26.4 (2.0)	
Household Income Level, %						< 0.0001
Below $25,000	40.8 (1.5)	25.5 (1.7)	37.5 (2.1)	52.4 (2.0)	62.6 (2.2)	
$25,000–$50,000	32.1 (1.1)	34.6 (2.0)	35.2 (1.6)	30.2 (1.9)	24.5 (2.1)	
Above $50,000	27.1 (1.6)	39.9 (2.5)	27.3 (2.0)	17.4 (1.6)	12.9 (1.8)	
***Cardiovascular***						
10-Year HF Risk, median (min, max)	1.6 (0.0, 82.7)	0.3 (0.0, 0.8)	1.5 (0.8, 2.7)	4.2 (2.7, 6.7)	10.8 (6.7, 82.7)	< 0.0001
QRS Duration, milliseconds, mean	98.3 (0.5)	96.3 (0.6)	97.5 (0.7)	98.5 (0.6)	103.2 (0.8)	< 0.0001
Systolic Blood Pressure, mmHg, mean	129.7 (0.4)	118.7 (0.6)	128.5 (0.6)	136.8 (0.6)	145.1 (0.8)	< 0.0001
Previous Myocardial Infarction, %	3.8 (0.3)	0.8 (0.4)	1.9 (0.5)	5.5 (0.9)	10.2 (1.0)	< 0.0001
Antihypertensive Medication, %	18.8 (0.7)	2.3 (0.6)	12.8 (1.1)	24.9 (1.5)	53.5 (1.8)	< 0.0001
***Metabolic***						
Body Mass Index, kg/m^2^, mean	27.4 (0.1)	25.5 (0.1)	28.0 (0.3)	28.3 (0.2)	29.2 (0.2)	< 0.0001
Total Cholesterol, mg/dL, mean	216.7 (0.9)	206.5 (1.4)	219.0 (1.5)	226.5 (1.6)	222.1 (1.2)	< 0.0001
HDL Cholesterol, mg/dL, mean	50.7 (0.5)	53.7 (0.6)	50.4 (0.7)	50.0 (0.6)	45.8 (0.7)	< 0.0001
Fasting Blood Glucose, mg/dL, mean	101.3 (0.8)	91.5 (0.5)	96.8 (1.0)	101.6 (1.1)	127.2 (2.2)	< 0.0001
Glucose-Lowering Medication, %	5.0 (0.3)	0.2 (0.1)	1.3 (0.3)	4.8 (0.7)	20.0 (1.8)	< 0.0001
***Mortality***						
Cardiovascular Disease Mortality, %	13.8 (0.6)	2.2 (0.4)	6.9 (0.8)	23.4 (1.4)	36.1 (1.5)	< 0.0001
All-cause Mortality, %	41.7 (1.3)	11.3 (1.2)	31.7 (1.7)	65.5 (1.9)	88.8 (1.1)	< 0.0001

* = percentages, means, medians, and measures of dispersion are estimated using U.S. population weights

† = measure of dispersion is standard error unless otherwise noted

*HDL* high-density lipoprotein, *Q1* quartile 1, *Q2* quartile 3, *Q3* quartile 3, *Q4* quartile 4, *PCP-HF* Pooled Cohort equations to Prevent Heart Failure

**Fig. 1:**
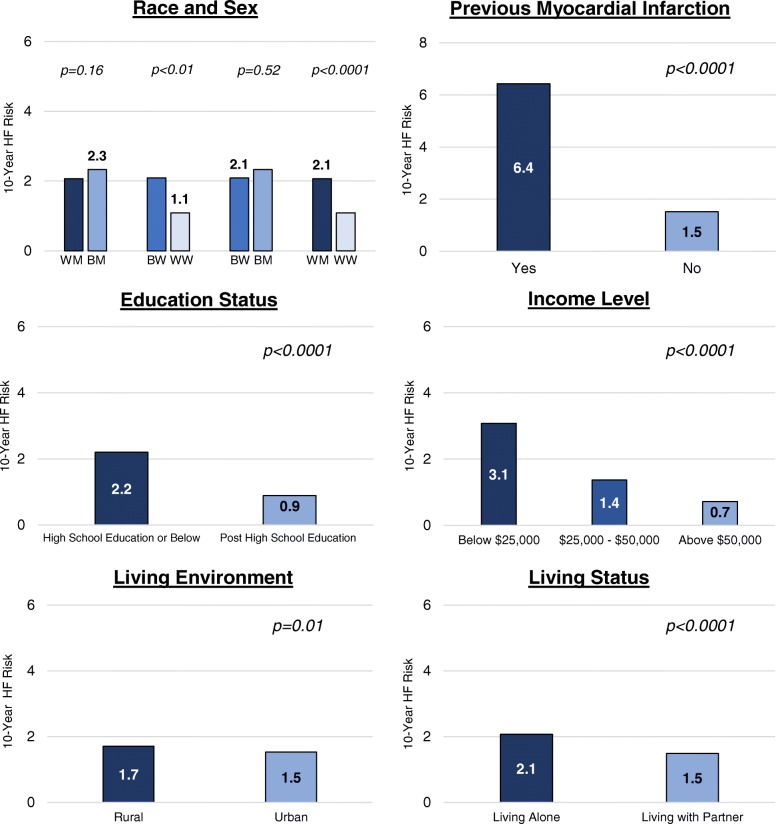
10-year PCP-HF Risk Score According to Clinical and Sociodemographic Variables. **Abbreviations**: BM = black men; BW = black women; PCP-HF = pooled cohort equations to prevent heart failure; WM = white men; WW = white women

The estimated median 10-year risk for incident HF was 1.6%. Figure 1 compares the average 10-year risk for incident HF by previous history of myocardial infarction and sociodemographic variables. Individuals self-reporting a previous myocardial infarction had a significantly higher predicted 10-year risk for HF compared with those who did not report a history of myocardial infarction (6.4% vs. 1.5%; *p* < 0.0001). Black women, adults living alone, living in rural settings, and adults with a high school education or lower had significantly higher PCP-HF risk scores compared to white women, adults living with partners, adults living in urban environments, and post high school graduates, respectively. White men also had a higher 10-year HF risk compared to white women (2.1% vs. 1.1%, p < 0.0001). Likewise, there were significant differences in HF risk according to annual income levels, with adults living in households with an annual income less than $25,000 having the highest estimated 10-year risk for incident HF (3.1%) among all income level strata.

**Fig. 2:**
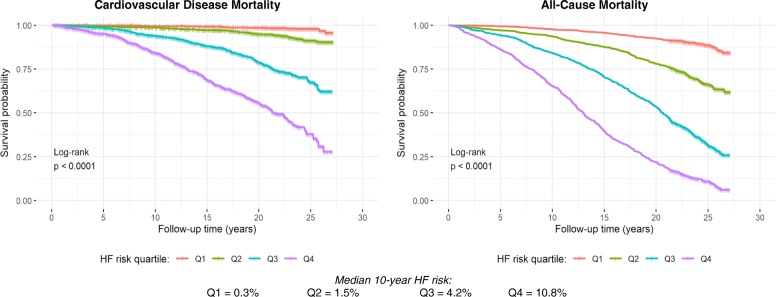
Kaplan Meier Estimates of Cardiovascular Disease and All-Cause Survival Probability According to PCP-HF Risk Score Quartiles. Abbreviations: PCP-HF = pooled cohort equations to prevent heart failure

Fig. 2 presents CVD and all-cause mortality survival probabilities stratified by PCP-HF risk score quartile over follow-up time. There were significant differences in survival among PCP-HF risk score quartiles (*p* < 0.0001) for both outcomes, and there was a shorter median survival time with each increasing PCP-HF risk score quartile. Among individuals with the highest 10-year risk for incident HF (quartile 4), median all-cause and CVD survival was 12.6 and 21.3 years, respectively.

**Fig. 3:**
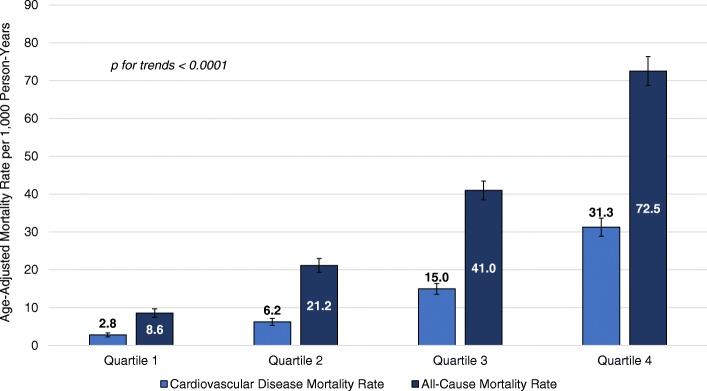
Age-adjusted Cardiovascular Disease and All-Cause Mortality Rates According to PCP-HF Risk Score Quartiles. Abbreviations: PCP-HF = pooled cohort equations to prevent heart failure

The association of the PCP-HF risk score with death due to CVD and all-causes is presented in Fig. 3 and Table 2. We observed a significant upward trend (*p* < 0.0001) in age-adjusted all-cause and CVD mortality rates from the lowest to highest quartile of PCP-HF risk score (Fig. 3). The age-adjusted CVD mortality rate for quartile 4 was 31.3 events per 1000 person-years, more than 11 times greater than the CVD mortality rate for individuals in the lowest quartile (2.8 events per 1000 person-years). Likewise, there was an 8-fold higher all-cause mortality rate when comparing the highest (72.8 events per 1000 person-years) versus the lowest PCP-HF risk score quartile (8.6 events per 1000 person-years). After adjusting for sociodemographic variables and previous history of myocardial infarction, there was a 58 and 38% higher risk of death due to CVD (HR = 1.58, 95% CI = 1.48, 1.70; *p* < 0.0001) and all-causes (HR = 1.38, 95% CI = 1.32, 1.46; p < 0.0001), for every two-fold increase in the PCP-HF risk score (Table 2).

**Table 2:** Hazard of Cardiovascular Disease and All-Cause Mortality According to Continuous PCP-HF Risk Score

Variable	Unadjusted		Model 2*		Model 3 †	
	Hazard Ratio (95% CI)	***P***-value	Hazard Ratio (95% CI)	***P***-value	Hazard Ratio (95% CI)	***P***-value
**Cardiovascular Disease Mortality**						
PCP-HF Risk Score ‡	2.03 (1.92, 2.15)	< 0.0001	1.64 (1.54, 1.75)	< 0.0001	1.58 (1.48, 1.70)	< 0.0001
(per doubling)	C-statistic = 0.795		C-statistic = 0.806		C-statistic = 0.812	
**All-Cause Mortality**						
PCP-HF Risk Score ‡	1.75 (1.67, 1.84)	< 0.0001	1.42 (1.35, 1.50)	< 0.0001	1.38 (1.32, 1.46)	< 0.0001
(per doubling)	C-statistic = 0.750		C-statistic = 0.765		C-statistic = 0.770	

*PCP-HF* pooled cohort equations to prevent heart failure

* = Adjusted for age, sex, and race

† = Adjusted for age, sex, race, living status, living environment, income level, educational attainment, and previous history of myocardial infarction

‡ = Log_2_ transformed

In unadjusted models, there was good discriminative ability of the PCP-HF risk score, and the risk score predicted CVD mortality (C-statistic = 0.795) to a better extent compared to all-cause mortality (C-statistic = 0.750). The addition of sociodemographic variables and history of myocardial infarction only modestly improved discriminative ability for both CVD (C-statistic = 0.812) and all-cause mortality (C-statistic = 0.770). The associations of all-cause and CVD-mortality with quartiles of the PCP-HF risk score were consistent with results using the linear, continuous PCP-HF risk score (Supplemental Table 3). In fully adjusted models, individuals in the highest quartile of PCP-HF risk score had more than a 9-fold higher risk of CVD mortality compared to individuals in the lowest quartile of predicted 10-year HF risk (HR = 9.71, 95% CI: 5.97, 15.81; p < 0.0001). All-cause and CVD-mortality discrimination were similar for models including the continuous and categorized PCP-HF risk score. Neither sex nor race significantly modified the association of the PCP-HF risk score with CVD or all-cause mortality.

## Discussion

This is the first study to characterize the 10-year risk for incident HF and assess the association of the recently derived PCP-HF risk score with CVD and all-cause mortality in a nationally representative sample of black and white men and women living in the United States. We found that the 10-year risk for incident HF varied according to social determinants of health, and groups with the highest HF risk included those with an annual household income below the poverty line, those living alone, as well as black participants. For each doubling of the PCP-HF risk score, individuals experienced a 58% higher hazard of CVD-associated death, suggesting that increases in HF risk, even among those at a low overall risk, are clinically significant for prevention purposes. We observed a similar, albeit smaller risk of all-cause death associated with the PCP-HF risk score.

We sought to evaluate differences in predicted HF risk across key sociodemographic strata. While both predicted and actual CVD events vary by education and living status, income level, and living environment [26, 27], the role of these sociodemographic factors in predicting incident HF remains largely unexplored. Low socioeconomic status, and its correlates, including low educational attainment, are independent risk factors for CVD events, including non-fatal myocardial infarction and sudden cardiac death, in both men and women [28]. Given that myocardial infarction is the primary cause of HF worldwide [29], and CVD remains the leading cause of death globally, it is likely that poverty and environmental factors, collectively known as social determinants of health, similarly influence the risk for incident HF. Pathways that may help explain these associations include lower access to quality care and a higher burden of smoking, obesity, and physical inactivity among individuals of lower socioeconomic status [30]. While the PCP-HF risk score is perhaps the most complete and generalizable HF risk score, changing demographic trends and the increasing income inequality gap [31] in the United States suggest that even this new 2019 score may not fully capture ethnic and racial diversity in HF risk prediction.

These data underscore the expanded utility of the PCP-HF risk score as a valuable epidemiologic and clinical risk-stratification tool to prevent HF and, more broadly, downstream CVD mortality in the United States. While there are similar components between the PCP-HF and other HF risk scores, the PCP-HF is the first HF risk score to be validated among a sample including young adults and a high proportion of black men and women. Likewise, the PCP-HF robustly predicts HF incidence using data readily available in the primary care setting. This is in contrast to other HF risk scores [9, 11] that include more complex biological variables, including N-terminal pro B-type natriuretic peptide, that are usually obtained more further downstream on the clinical HF workup. Our study lays the foundation for future endeavors that compare the PCP-HF risk score with current general CVD risk scores to assess whether patient motivation and primary prevention change based on the estimated risk across different models, even though the scores predict different clinical outcomes. For example, a patient at intermediate CVD risk may be more motivated by a 25–30% predicted risk of incident HF versus a 15% predicted risk of an CVD event over the next 10 years.

Our study builds on previous reports regarding differences in HF incidence and risk factors. Analyses from the Atherosclerosis Risk in Communities (ARIC) study demonstrate that black males had the highest HF incidence, 9.1 cases per 1000 person-years, compared to black women, white men, and white women [32]. Likewise, the Coronary Artery Risk Development in Young Adults (CARDIA) study reported that black adults under the age of 50 years have a 20-fold higher incidence of early onset HF, compared to whites [33]. Consistent with these observations, we found that black men had the highest predicted 10-year risk for incident HF, at 2.3%, followed by black women, 2.1%, white men, 2.1%, and white women, 1.1%. These findings may be partially explained by considering the various different forms of HF. HF is a heterogenous disease with several different risk factors and disease subtypes, including heart failure with reduced ejection fraction (HFrEF) as well as heart failure with preserved ejection fraction (HFpEF). The lifetime risk of HFrEF is higher in men compared to women, while the lifetime risk of HFpEF is higher in whites versus blacks [34]. Furthermore, female sex is an established etiological risk factor for HFpEF [35].

In addition to documenting a higher risk of death due to CVD and all causes across increasing PCP-HF risk, we also identified discrete score cut offs that that may be used by clinicians to improve HF risk stratification. Similar to the Pooled Cohort Equations CVD risk score [36], there may low-, intermediate-, and high-risk zones for HF that could potentially guide clinical decision making. Based on observed CVD mortality rates across PCP-HF risk quartiles, a measured risk falling within quartiles 1 and 2 (0 to 2.7%) appears to confer a low risk for a future CVD-attributed death, or less than 7 events per 1000 person years. On the other hand, calculated PCP-HF risk scores between 2.7 and 6.7%, quartile 3, and greater than 6.7%, quartile 4, were associated with an intermediate- (15 events per 1000 person years) and high-risk (32 events per 1000 person years) for CVD mortality. Future studies are warranted to confirm our findings and improve the accuracy and definition of clinically relevant PCP-HF risk score categories.

Increases in population diversity, aging of the baby boomer population, and a rapidly increasing HF epidemic [1–3] have warranted urgent public health action to improve HF prevention. Our study, using a nationally representative sample of black and white men and women across different sociodemographic subgroups, serves to inform such strategies. Thus, utilization of NHANES data and HF risk estimation through sex- and race-specific equations enhances the generalizability of our findings and highlights the notable lack of race/ethnic-specific HF risk prediction tools available.

Other important strengths of this study include long follow-up time as well as the use of hard endpoints, including death due to CVD or all causes. On the other hand, we were not able to differentiate the association of the PCP-HF risk score with specific types of CVD-death (ex: HF, acute myocardial infarction, hypertensive heart disease, arrhythmia, etc.) due to the lack of disaggregated cause of death data. Availability of such data would have enabled us to further explore the discriminative ability of the PCP-HF risk score specifically associated with HF mortality. Another important consideration is that the PCP-HF risk score does not distinguish between predicted 10-year incidence of HFrEF versus HFpEF. This latter limitation is an essential factor to address in future studies given the changing epidemiological landscape of HF, as the prevalence of HFpEF is growing and now accounts for at least one-half of all HF cases [37].

## Conclusions

Here, we have identified a strong association between the PCP-HF risk score and CVD and all-cause mortality in a large, representative sample of United States adults. Individuals with the highest 10-year predicted risk for incident HF include those who reported a previous history of myocardial infarction, black participants, as well as persons with low educational attainment or an annual household income below $25,000. Thus, social determinants of health may influence predicted HF risk and mortality, and future predictive equations should strive to capture these measures. Most importantly, these findings highlight the expanded utility of the PCP-HF risk score and suggest that its more formal use at both the clinical and population health levels may help improve primary CVD prevention in the United States.

## Supplementary information

**Additional file 1: Table S1.** Sex- and race-specific equation parameters for estimation of 10-year HF risk. **Table S2.** Person-Time and Crude Number of Deaths According to PCP-HF Risk Score Quartile. **Table S3.** Hazard of Cardiovascular Disease and All-Cause Mortality According to PCP-HF Risk Score Quartiles.

## Data Availability

All data used for the current manuscript is online and currently available through NHANES III datasets (https://wwwn.cdc.gov/nchs/nhanes/nhanes3/DataFiles.aspx) and the survey’s linked mortality dataset (https://www.cdc.gov/nchs/data-linkage/mortality.htm). This study did not use restricted access data.

## Abbreviations

BMI: Body mass index
CVD: Cardiovascular disease;
HDL: High-density lipoprotein
HF: Heart failure
HFpEF: Heart failure with preserved ejection fraction
HFrEF: Heart failure with reduced ejection fraction
NHANES: National health and nutritional examination survey
PCP-HF: Pooled cohort equations to prevent heart failure
