# Supplementary material for: Pooled cohort equations heart failure risk score predicts cardiovascular disease and all-cause mortality in a nationally representative sample of US adults
Source: BMC Cardiovasc Disord. 2020 Apr 25;20:202. doi: 10.1186/s12872-020-01485-2 (PMC7183589; doi:10.1186/s12872-020-01485-2)
Supplement: Supplementary file 1 — Additional file 1: Table S1. Sex- and race-specific equation parameters for estimation of 10-year HF risk. Table S2. Person-Time and Crude Number of Deaths According to PCP-HF Risk Score Quartile. Table S3. Hazard of Cardiovascular Disease and All-Cause Mortality According to PCP-HF Risk Score Quartiles. [file 12872_2020_1485_MOESM1_ESM.docx]

| **Supplement Table 1.** Sex- and race-specific equation parameters for estimation of 10-year HF risk | | | | | | | | | | | | | |
| --- | --- | --- | --- | --- | --- | --- | --- | --- | --- | --- | --- | --- | --- |
|  | **White Men (n=2302)** | | | **White Women (n=2421)** | | | **Black Men (n=754)** | | | **Black Women (n=856)** | | | |
|  | **Coefficient*** | **Mean Sample Value** | **Coefficient x Value** | **Coefficient*** | **Mean Sample Value** | **Coefficient x Value** | **Coefficient*** | **Mean Sample Value** | **Coefficient x Value** | **Coefficient*** | **Mean Sample Value** | **Coefficient x Value** |  |
| Ln age, years | 41.94101 | 3.981888 | 167.004404 | 20.54973 | 3.995292 | 82.1021719 | 2.88334 | 3.950039 | 11.38930545 | 51.75667 | 3.956840 | 204.7928621 |  |
| Ln age, squared | -0.88115 | 15.894273 | -14.0052387 | N/A | N/A | N/A | N/A | N/A | N/A | N/A | N/A | N/A |  |
| Ln treated systolic blood pressure, mmHg | 1.030508 | 4.945331 | 5.09620316 | 12.94937 | 4.941164 | 63.9849609 | 2.31106 | 4.962534 | 11.46871383 | 28.97791 | 4.955850 | 143.6101753 |  |
| Ln age x Ln treated systolic blood pressure | N/A | N/A | N/A | -2.96923 | 20.316412 | -60.3241 | N/A | N/A | N/A | -6.59777 | 20.038534 | -132.2096385 |  |
| Ln untreated systolic blood pressure, mmHg | 0.91252 | 4.846408 | 4.42244423 | 11.86273 | 4.820778 | 57.1875878 | 2.17229 | 4.866329 | 10.57107782 | 28.1853 | 4.834801 | 136.2703166 |  |
| Ln age x Ln untreated systolic blood pressure | N/A | N/A | N/A | -2.72538 | 19.142069 | -52.169412 | N/A | N/A | N/A | -6.42425 | 19.113140 | -122.7875896 |  |
| Current smoker (0=no, 1=yes) | 0.73839 | 0 or 1 | 0 or 0.73839 | 11.01752 | 0 or 1 | 0 or 11.01752 | 1.65337 | 0 or 1 | 0 or 1.65337 | 0.76532 | 0 or 1 | 0 or 0.76532 |  |
| Ln age x current smoker | N/A | N/A | N/A | -2.50777 | 0 or 1 | 0 or -2.50777 | -0.24665 | 0 or 1 | 0 or -0.24665 | N/A | N/A | N/A |  |
| Ln treated glucose, mg/dL | 0.90072 | 5.052547 | 4.55093013 | 1.04503 | 5.171867 | 5.40475617 | 0.64704 | 5.151292 | 3.333091976 | 0.96695 | 5.268461 | 5.094338364 |  |
| Ln untreated glucose, mg/dL | 0.77805 | 4.577495 | 3.56151998 | 0.91807 | 4.535281 | 4.16370543 | 0.57891 | 4.574070 | 2.647974864 | 0.79561 | 4.559626 | 3.627684042 |  |
| Ln total cholesterol, mg/dL | 0.49323 | 5.347526 | 2.63756025 | N/A | N/A | N/A | N/A | N/A | N/A | 0.32646 | 5.353074 | 1.747564538 |  |
| Ln HDL-C, mg/dL | -0.43683 | 3.747008 | 1.6368055 | -0.07455 | 3.94000 | -0.293727 | -0.80691 | 3.990176 | 3.219712916 | N/A | N/A | N/A |  |
| Ln BMI, kg/m^2^ | 37.21577 | 3.297969 | 122.736456 | 1.32948 | 3.277853 | 4.35784001 | 1.16289 | 3.377444 | 3.927595853 | 21.24763 | 3.377444 | 71.76268046 |  |
| Ln age x Ln BMI | -8.83278 | 13.131618 | -115.988693 | N/A | N/A | N/A | N/A | N/A | N/A | -5.00068 | 13.362623 | -66.82220158 |  |
| Ln QRS duration, ms | 0.63224 | 4.624964 | 2.92408724 | 1.06089 | 4.541116 | 4.81762455 | 0.72646 | 4.540999 | 3.298854134 | 1.27475 | 4.540999 | 5.788638475 |  |
| Mean coefficient x Sample Value (IndCV) | N/A | N/A | 171.974102 | N/A | N/A | 100.116220 | N/A | N/A | 29.044386 | N/A | N/A | 234.816152 |  |
| Mean coefficient x Equation Value (MeanCV) | N/A | N/A | 171.59 | N/A | N/A | 99.7321 | N/A | N/A | 28.7369 | N/A | N/A | 233.978 |  |
| Baseline Survival (S_0_) | N/A | N/A | 0.98752 | N/A | N/A | 0.99348 | N/A | N/A | 0.98295 | N/A | N/A | 0.99260 |  |
| **Mean Estimated 10-year risk of HF (%)** | N/A | N/A | 4.566913% | N/A | N/A | 3.065205% | N/A | N/A | 4.172934% | N/A | N/A | 4.427866% |  |
| **Median Estimated 10-year risk of HF (%)** | N/A | N/A | 2.070281% | N/A | N/A | 1.085351% | N/A | N/A | 2.327478% | N/A | N/A | 2.086609% |  |
| * = Provided from previously derived PCP-HF risk score derivation analyses  Predicted 10-year HF risk = 1 – S_0_ ^e(IndCV – MeanCV)^ | | | | | | | | | | | | | |

| **Supplemental Table 2**. Person-Time and Crude Number of Deaths According to PCP-HF Risk Score Quartile | | | | | |
| --- | --- | --- | --- | --- | --- |
|  | **Overall**  **(n=6333)** | **PCP-HF Q1**  **(n=1583)** | **PCP-HF Q2**  **(n=1583)** | **PCP-HF Q3**  **(n=1584)** | **PCP-HF Q4**  **(n=1583)** |
| Person-time, years, median (Q1, Q3) | 22.3 (16.7, 24.5) | 23.7 (22.1, 25.3) | 22.9 (21.3, 24.9) | 20.6 (13.6, 23.6) | 12.9 (7.9, 18.9) |
| Cardiovascular Disease Mortality, n | 1116 | 51 | 142 | 373 | 600 |
| All-Cause Mortality, n | 3178 | 235 | 522 | 1035 | 1386 |
| PCP-HF = pooled cohort equations to prevent heart failure | | | | | |
|  |  |  |  |  |  |

| **Supplemental Table 3.** Hazard of Cardiovascular Disease and All-Cause Mortality According to PCP-HF Risk Score Quartiles | | | | | | |
| --- | --- | --- | --- | --- | --- | --- |
| **Variable** | **Unadjusted** | | **Model 2 *** | | **Model 3** † | |
|  | **Hazard Ratio (95% CI)** | ***P*-value** | **Hazard Ratio (95% CI)** | ***P*-value** | **Hazard Ratio (95% CI)** | ***P*-value** |
| **Cardiovascular Disease Mortality** |  |  |  |  |  |  |
| PCP-HF Risk Score ‡ |  |  |  |  |  |  |
| Quartile 1 | Ref | - | Ref | - | Ref | - |
| Quartile 2 | 3.63 (2.27, 5.81) | <0.0001 | 2.13 (1.33, 3.40) | <0.01 | 2.02 (1.27, 3.22) | <0.01 |
| Quartile 3 | 16.50 (10.89, 24.98) | <0.0001 | 5.83 (3.61, 9.43) | <0.0001 | 5.27 (3.26, 8.54) | <0.0001 |
| Quartile 4 | 42.66 (28.22, 64.49) | <0.0001 | 11.34 (7.03, 18.30) | <0.0001 | 9.71 (5.97, 15.81) | <0.0001 |
|  | C-statistic = 0.776 | | C-statistic = 0.802 | | C-statistic = 0.808 | |
| **All-Cause Mortality** |  |  |  |  |  |  |
| PCP-HF Risk Score ‡ |  |  |  |  |  |  |
| Quartile 1 | Ref | - | Ref | - | Ref | - |
| Quartile 2 | 3.19 (2.67, 3.81) | <0.0001 | 1.88 (1.54, 2.29) | <0.0001 | 1.81 (1.48, 2.22) | <0.0001 |
| Quartile 3 | 8.69 (6.89, 10.99) | <0.0001 | 3.05 (2.34, 3.99) | <0.0001 | 2.82 (2.15, 3.70) | <0.0001 |
| Quartile 4 | 19.69 (15.53, 24.89) | <0.0001 | 5.13 (3.95, 6.67) | <0.0001 | 4.57 (3.47, 6.03) | <0.0001 |
|  | C-statistic = 0.731 | | C-statistic = 0.761 | | C-statistic = 0.767 | |
| PCP-HF = pooled cohort equations to prevent heart failure  * = Adjusted for age, sex, and race  † = Adjusted for age, sex, race, living status, living environment, income level, educational attainment, and previous history of myocardial infarction  ‡ = Log_2_ transformed | | | | | | |
